# Carbon ion radiotherapy as definitive treatment in non-metastasized pancreatic cancer: study protocol of the prospective phase II PACK-study

**DOI:** 10.1186/s12885-020-07434-8

**Published:** 2020-10-01

**Authors:** Jakob Liermann, Patrick Naumann, Adriane Hommertgen, Moritz Pohl, Meinhard Kieser, Juergen Debus, Klaus Herfarth

**Affiliations:** 1 Department of Radiation Oncology, Heidelberg University Hospital, Im Neuenheimer Feld 400, 69120 Heidelberg, Germany; 2 Heidelberg Institute of Radiation Oncology (HIRO), Im Neuenheimer Feld 400, 69120 Heidelberg, Germany; 3 Heidelberg Ion-Beam Therapy Center (HIT), Im Neuenheimer Feld 450, 69120 Heidelberg, Germany; 4 National Center for Tumor Diseases (NCT), Im Neuenheimer Feld 460, 69120 Heidelberg, Germany; 5 Clinical Cooperation Unit Radiation Oncology, German Cancer Research Center (DKFZ), Im Neuenheimer Feld 280, 69120 Heidelberg, Germany; 6 German Cancer Consortium (DKTK), partner site Heidelberg, German Cancer Research Center (DKFZ), Im Neuenheimer Feld 280, 69120 Heidelberg, Germany; 7 Institute of Medical Biometry and Informatics, University of Heidelberg, Im Neuenheimer Feld 130.3, 69120 Heidelberg, Germany

**Keywords:** Carbon ion radiotherapy, Particle therapy, Pancreatic cancer

## Abstract

**Background:**

Radiotherapy is known to improve local tumor control in locally advanced pancreatic cancer (LAPC), although there is a lack of convincing data on a potential overall survival benefit of chemoradiotherapy over chemotherapy alone. To improve efficacy of radiotherapy, new approaches need to be evolved. Carbon ion radiotherapy is supposed to be more effective than photon radiotherapy due to a higher relative biological effectiveness (RBE) and due to a steep dose-gradient making dose delivery highly conformal.

**Methods:**

The present Phase II PACK-study investigates carbon ion radiotherapy as definitive treatment in LAPC as well as in locally recurrent pancreatic cancer. A total irradiation dose of 48 Gy (RBE) will be delivered in twelve fractions. Concurrent chemotherapy is accepted, if indicated. The primary endpoint is the overall survival rate after 12 months. Secondary endpoints are progression free survival, safety, quality of life and impact on tumor markers CA 19–9 and CEA. A total of twenty-five patients are planned for recruitment over 2 years.

**Discussion:**

Recently, Japanese researches could show promising results in a Phase I/II-study evaluating chemoradiotherapy of carbon ion radiotherapy and gemcitabine in LAPC. The present prospective PACK-study investigates the efficacy of carbon ion radiotherapy in pancreatic cancer at Heidelberg Ion Beam Therapy Center (HIT) in Germany.

**Trial registration:**

The trial is registered at ClinicalTrials.gov: NCT04194268 (Retrospectively registered on December, 11th 2019).

## Background

Pancreatic cancer is a highly lethal cancer. Despite all effort, the five-year overall survival rate is currently at merely 5–10% [1]. In 2018, the global mortality to incidence ratio was at 94% [2]. So far, operation is the solely curative therapy option but even the postoperative five-year overall survival rate is at only 20% [3]. Often diagnosed in a late stage of disease, approximately one third of all patients initially present with non-metastasized but inoperable locally advanced pancreatic cancer (LAPC) [4]. In the last decades, different neoadjuvant and definitive treatment schemes were evaluated and optimal care is still in discussion. In 2016, Hammel et al. published the prospective, international LAP 07 trial, in which patients underwent chemotherapy and only those who did not show tumor progression after 4 months were randomized in further treatment with chemotherapy (*n* = 136) or chemoradiotherapy (*n* = 133) [5]. Although, there was no difference in overall survival, chemoradiotherapy was shown to improve local tumor control compared to chemotherapy alone. Altogether, the role of photon radiotherapy in LAPC is still inconclusive. Recently, Icaobuzio-Donahue et al. could show that local tumor progression is related with approximately one third of all pancreatic cancer deaths [6], highlighting the necessity of effective treatment schemes for patients suffering from LAPC and from non-metastasized locally recurrent pancreatic cancer. To improve efficacy of radiotherapy in pancreatic cancer therapy, different approaches are made. On the one hand, radiation dose prescription is limited due to the organs at risk (OAR) nearby. The duodenum and the stomach as well as the small intestine in total are sensitive tissues with a high risk of ulceration or perforation in case of overdosing [7, 8]. Higher conformity and consequently higher doses in the target volume without the risk of OAR-overdosing could try to be reached by modern radiation techniques. On the other hand, there are pancreatic cancer-tissue specific properties that could explain the missing effect on overall-survival of radiotherapy. Predominantly, hypoxic tissue is relatively resistant to photon radiotherapy because photon radiation damage is mainly induced by the production of reactive oxygen species and pancreatic cancer is known to be hypoxic [9]. An approach to overcome this gap of efficacy of photon irradiation is the use of particle therapy, which induces more direct radiation damage to the deoxyribonucleic acid (DNA) due to the known higher linear energy transfer (LET) corresponding with a higher relative biological effectiveness (RBE). Hence, particle therapy is supposed to be more effective in hypoxic tumors. In vitro, carbon ion radiotherapy shows higher RBE-values in pancreatic cancer than those commonly known from protons [10–12]. Therefore, it could be an effective treatment scheme for patients suffering from LAPC or non-metastasized locally recurrent pancreatic cancer.

Recently, Shinoto et al. presented promising results of a prospective Phase I/II dose-escalation study investigating carbon ion radiotherapy in combination with gemcitabine [13]. Seventy-two patients were irradiated from 43.2 Gy (RBE) up to 55.2 Gy (RBE) in twelve fractions over 3 weeks. The described median overall survival (OS) was at 19.6 months. There was a tendency to improved results in the higher-dosed irradiations. The twelve-months overall survival rate was at 73%. The CT-based local control rate after 2 years was at 83%. Apart from six cases of grade 3 anorexia, only one clearly radiation-induced grade 3 toxicity was observed: a gastrointestinal ulceration.

In our facility, Combs et al. retrospectively evaluated the oncological response of 57 patients suffering from LAPC receiving neoadjuvant chemoradiotherapy with photons (intensity modulated radiotherapy: IMRT) and gemcitabine [14]. The dose prescription was divided in a primary treatment plan (including tumor + lymphatic drainage) of a median total dose of 45.0 Gy in single doses of 1.8 Gy and in a boost irradiation (including tumor only) up to a median total dose of 54.0 Gy in single doses of median 2.2 Gy. The observed median OS was at 11 months and the twelve-months OS rate was at 36%. The local progression free survival (LPFS) rate after 2 years was at 13%. Toxicity rates were low.

Compared to the prospective patient cohort treated with photon radiotherapy of Hammel et al. [5] and especially compared to our in-house data from Combs et al. [14], the findings of Shinoto et al. seem very promising (see Table 1). To our knowledge, there is no prospective trial investigating carbon ion radiotherapy in locally recurrent pancreatic cancer. Retrospective data of carbon ion radiotherapy in Japanese patients suffering from locoregional recurrent pancreatic cancer are very promising, too. Kawashiro et al. observed a median overall survival of 25.9 months in this patient cohort [15]. Our in-house data from Habermehl et al. showed a median overall survival of 16.1 months of locally recurrent pancreatic cancer patients treated with photon radiotherapy [16].

**Table 1:** Comparison of a retrospective analysis of neoadjuvant treated pancreatic cancer patients with photon radiotherapy (IMRT) in our facility by Combs et al. [14] with a Phase I/II prospective trial investigating carbon ion radiotherapy in pancreatic cancer by Shinoto et al. [13]

	Author	Number of patients	Number of fractions	Total dose in Gy (RBE)	Single dose in Gy (RBE)	1 J OS [%]	2 J OS [%]	Median OS [months]	Concurrent gemcitabine
Photons	**Combs et al.**	57	25	54	2.2	36	8	11	300 mg/m^2^, weekly
Carbon ions	**Shinoto et al.**	72	12	43.2–55.2	3.6–4.6	73	35	19,6	400–1000 mg/m^2^; days 1, 8, 15

Concerning carbon ion radiotherapy, different facilities use different RBE-calculation models as well as different radiation techniques (active scanning vs. passive scattering) [17]. Furthermore, apart from the results of Shinoto et al., there are no other published data of prospective trials evaluating carbon ion radiotherapy in pancreatic cancer. Therefore, the present PACK-study will investigate carbon ion radiotherapy in patients suffering from LAPC or locally recurrent pancreatic cancer at HIT, Germany.

## Methods/design

### Aim of the study and endpoints

The aim of the present trial is to investigate the efficacy of carbon ion radiotherapy in localized pancreatic cancer. Therefore, the primary endpoint is the OS rate after 12 months (1y-OS), the 12 months time period starts with the first day of radiotherapy.

Secondary endpoints are the progression free survival rate after 12 months, incidence of non-hematological NCI CTC AE V5.0 grade III-V toxicity, quality of life evaluated by standardized questionnaires (EORTC QLQ-C30 and EORTC QLQ-PAN26) and the course of the tumor markers CA 19–9 and CEA during follow-up.

### Recruitment

The planned trial is a single-center study due to the rarity of carbon ion radiation facilities. Hence, all patients will be recruited in the Department of Radiation Oncology, Heidelberg University Hospital, Germany. A total number of 25 patients is planned to be recruited into the study. Based on our clinical experience, the recruitment phase is calculated to last approximately 2 years and will be finished in December 2021.

### Eligibility – inclusion criteria

- Histologically confirmed, inoperable primary or locally recurrent pancreatic cancer
- Written informed consent (must be available before enrollment in the trial)
- Karnofsky performance score > 60 or ECOG-status 0/1 (at least: patient should be able to take care of himself, although daily-life activity or work is not possible)
- Age ≥ 18 years

### Eligibility – exclusion criteria

- No clear difference between tumor edge and upper gastrointestinal tract in baseline imaging
- Extensive lymphatic metastases
- Disability of subject to understand character and individual consequences of the clinical trial
- Distant metastases
- Previous radiotherapy of the upper abdomen
- Active medical implants (e.g. pacemaker, defibrillator), contradicting radiotherapy at HIT
- Participation in another clinical study or observation period of competing trials, respectively

Comment: Patients treated with prior systemic therapy are not excluded from study participation.

### Radiotherapy

#### Planning CT

Each patient will be positioned in supine position. A contrast enhanced CT of the upper abdomen will be done in different phases (native, arterial, venous, late-venous). The slice thickness is set at 3 mm. 4D-CT-imaging will be performed to take account of respiratory movement of the target and the OAR. The patient is adviced to fast for at least 3 h before planning procedure and before each radiotherapy fraction.

#### Contouring

Macroscopic tumor in the planning CT and – if available – in other imaging modalities (MRI, PET/CT) will be defined as Gross Tumor Volume (GTV). The Clinical Target Volume (CTV) will be defined as an expansion of 6 mm of the GTV. Within CTV-definition, anatomic boundaries (e.g. bone) will be respected, if not infiltrated. The macroscopic tumor burden at the time of the planning imaging is the crucial information. Pre-chemotherapy volumes will not be included in the CTV per se, although all patient-based data will be studied to define an individual CTV. If patients responded to prior systemic therapy, the pre-systemic therapy GTV should be respected in the CTV definition. The Internal Target Volume (ITV) will be defined as CTV with an individual expansion according to the respiratory movement in the 4D-CT. The Planning Target Volume (PTV) will be defined as an expansion of 5 mm of the ITV. Kidneys, upper gastrointestinal tract, liver and spinal cord will be contoured as OAR.

#### Radiotherapy planning

The treatment will be planned using the treatment planning software (TPS) RT-Planning (Siemens, Erlangen, Germany). Included is a biological plan optimization using the local effect model (LEM) I, established at GSI Helmholtzzentrum für Schwerionenforschung and at HIT [18]. The underlying clinical α/β-ratio for LEM I is set at 5 Gy for the ITV and at 2 Gy for the OAR (also, if there is an OAR-overlap with the ITV). The total dose of 48 Gy (RBE) correspond to an equivalent dose at 2 Gy (EQD2) of 61.7 Gy and to a biological equivalent dose (BED) of 86.4 Gy using an α/β-ratio of 5 Gy. If clinically possible, 95% of the PTV should be covered by 95% of the prescribed dose. A homogenous dose delivery is intended.

#### Dose prescription and irradiation

A total dose of 48 Gy (RBE) will be applied in 12 fractions with a single dose of 4 Gy (RBE). 5–6 fractions will be irradiated per week, one fraction per day (2–2.5 weeks). The dose will be prescribed to the ITV. Radiotherapy will be done using an intensity-controlled rasterscanning system for beam application.

#### Dose constraints

The kidney volume receiving more than 24 Gy (RBE) must not exceed 20% of the total kidney volume. The accepted maximum dose in the spinal cord is defined at 36 Gy (RBE). The maximum dose in the upper gastrointestinal is 43.2 Gy (RBE). This volume is defined on the native CT scan. Additionally, gastrointestinal movement is evaluated in the 4D-CT and in the different contrast-enhanced phases of the performed CT. Adding this movement information to the upper gastrointestinal, a separate “GI movement” volume is defined. In parts of this “GI movement” volume that do overlap with the ITV, a maximum dose of 45.6 Gy (RBE) is accepted. If this cannot be reached, decreased dosage in the ITV has to be accepted. The liver should be irradiated as low as reasonably achievable (ALARA).

#### Image-guidance

Orthogonal X-rays will be used as image-guidance prior to the irradiation. Bones as well as clips (in cases of locally recurrent pancreatic cancer) are used for alignment. As there still is a risk of geometric miss, additional CT scans are performed regularly to reassure the calculated dose distribution in the target volumes and the OARs. If necessary, the radiation plan will be adjusted to the current CT scan.

### Chemotherapy

Concurrent chemotherapy is not part of the study protocol. If indicated, the patients will be treated according to the in-house standard operating procedure using gemcitabine 300 mg/m^2^ body surface once a week as concurrent systemic therapy. This chemotherapy scheme is comparable to the in-house photon radiotherapy data of Combs et al. [14]. We expect most of the patients to present with indication of concurrent systemic therapy. Chemotherapy continuation is at the discretion of the treating medical oncologist. Systemic therapy history will be evaluated in detail for each participating patient.

### Follow-up

The oncological follow-up starts 3 months after the first day of radiotherapy. Further follow-ups will be performed at months 6, 9, 12 and 15. Elements of each follow-up examination as well as a general trial schedule are shown in Table 2. Patients lost to follow-up will be censored.

**Table 2:** Trial schedule, *imaging is done by CT or MRI or PET/CT, RT = radiotherapy

	Inclusion in trial	Planning of RT (week -2)	RT (week 0–2)	End of RT (week 2)	Follow-up 1 (month 3)	Follow-up 2 (month 6)	Follow-up 3 (month 9)	Follow-up 4 (month 12)	Follow-up 5 (month 15)
Eligibility	x								
Written informed consent	x								
Medical History (incl. Histology)	x			x	x	x	x	x	x
Karnofsky performance score	x			x	x	x	x	x	x
Height	x								
Weight	x			x	x	x	x	x	x
Blood sample (CA 19–9, CEA)	x				x	x	x	x	x
QLQ-C30 and QLQ-PAN26	x			x	x	x	x	x	x
Toxicity (NCI CTC AE V5.0)	x			x	x	x	x	x	x
Staging imaging* (< 2 months old)	x								
Imaging* of the upper abdomen					x	x	x	x	x
CT of the chest						x		x	
Planning CT		x							
Image-guidance during RT			x						

### Assessment of efficacy

Complete remission (CR), partial remission (PR), stable disease (SD) and progressive disease (PD) will be defined for the target and in non-target lesions at each follow-up according to the RECIST 1.1 -criteria [19].

The OS is defined as time from the start of radiotherapy until reported death due to any cause. To assess efficacy, we use the 1y-OS rate, which is the Kaplan Meier estimate at 1 year.

The PFS is defined from the start of radiotherapy until any tumor progression or death due to any cause.

The LPFS will be evaluated from the start of radiotherapy until progression of the irradiated target based on the RECIST criteria or death. Any progression out of the high-dose radiation field will not be defined as local progression and will be denominated as regional progression, if nearby.

Blood samples will be collected at enrollment of the trial and at each follow-up (see Table 2). The course of the tumor markers CA 19–9 and CEA will be evaluated.

### Toxicity

Toxicity will be evaluated using the International Common Terminology Criteria for Adverse Events of the National Cancer Institute (NCI CTC AE), Version 5. Any new symptom will be imposed. Furthermore, the following symptoms will be actively investigated at each follow-up: nausea, fatigue, fever, abdominal pain, diarrhea, weight loss, obstipation, duodenal hemorrhage, duodenal ulcer, dyspepsia, ascites, dermatitis.

### Safety

An independent data safety monitoring board (DSMB) will continuously supervise recruitment, adverse events and quality of the data. The aim of the board is to ensure safety of the patients and ethical correctness during the trial. The DSMB can give recommendations concerning changing, continuing or ending the trial. It consists of experts of the field.

### End of study

The regular end of the trial is set after the last follow-up 15 months after the start of radiotherapy. An irregular individual end could be provoked by the patient. A general end of study could be provoked by the appearance of one grade 5 toxicity, by two grade 4 toxicities in a row, by five grade 3 toxicities in a row, by new data contradicting the present trial, by inadequate recruitment or by the Principal Investigator if risks are deemed too high.

### Sample size

The primary endpoint of this single-arm trial is the 1y-OS rate, which was chosen to compare the study results with the 1y-OS results (36%) from Combs et al., (photons) [14] and to reproduce the 1y-OS results (73%) reported by Shinoto et al., (carbon ions) [13].

A planned sample size of 25 patients accounts for an assumed drop-out rate of 10% (2–3 patients) within 1 year after treatment start. With 3 drop-outs and 6 deaths within the first 12 months, one would end up with a 1y-OS rate of 72.7% and a 95%-confidence interval of [0.541, 0.913] (80%-confidence interval of [0.606, 0.849]). To represent the most conservative approach, the drop-outs are assumed to all take place before the first patient dies.

The sample size allows to compare the trial results with the in-house results from Combs et al. [14] using the confidence interval for the 1y-OS rate and enables a possible reproduction of the promising results from Shinoto et al. [13]

### Statistical analysis

The primary endpoint will be analyzed using the non-parametric Kaplan-Meier estimate. The 1y-OS rate will be presented along with the 80% confidence interval and the 95% confidence interval. Results from secondary endpoints will be presented using methods of descriptive data analysis. The appropriate methods are chosen based on the scale and characteristics of the variables. The incidence of grade 3/4 NCI-CTC-AE toxicities are described by counts, which are presented as absolute and relative frequencies. Interim analyses with pre-specified statistical stopping rules are not planned, because a sample size of 25 patients is necessary to obtain the required amount of information and power regarding the primary objective of this trial.

### Study population

The primary analysis will be conducted on a modified intention-to-treat population which is defined as all patients which are compliant with the in- and exclusion criteria and who received at least 1 week of study treatment. Censoring at the end of the follow-up period is considered to be administrative. Censoring implicitly assumes that the patient would have behaved as the other patients if he had been observed. Drop-outs are considered to be non-informative and censored at the last observation. In sensitivity analyses, the primary analysis is conducted on a per-protocol population, containing all patients who completely underwent the planned therapy. Safety analysis are conducted on the safety population which contains all enrolled patients who started therapy.

### Data management

Data management is handled according to the German law. Collected data will be pseudonymized. All documentations will be archived for 30 years.

### Ethical and legal issues

The study protocol, the patient information sheet and the declaration of informed consent form were evaluated by the Ethics committee of the Medical faculty of the University of Heidelberg (S-203/2019). Principles of Good Clinical Practice (GCP) guidelines are supervised by the above mentioned independent DSMB. All patients must declare their informed consent to participate in the trial in written form before enrollment. The trial is also approved by the Federal Radiation Safety Agency in Germany (Z5–22464/2019–102-G).

## Discussion

The prospective data of Shinoto et al. [13] investigating carbon ion radiotherapy in LAPC and also as neoadjuvant treatment scheme in potentially resectable pancreatic cancer [20] are very promising and attracted attention in the field of research. Currently, several studies are ongoing to investigate a benefit of carbon ion radiotherapy in pancreatic cancer [21]. In Italy, the multicenter Phase II PIOPPO-trial (trial registration at ClinicalTrials.gov: NCT03822936) [22] is evaluating carbon ion radiotherapy as neoadjuvant therapy in resectable and borderline resectable pancreatic cancer. The University of Texas Southwestern Medical Center, USA, and the National Institute of Radiological Sciences, Japan, collaborate in the Phase III CIPHER-trial to further evaluate carbon ion radiotherapy in LAPC (trial registration at ClinicalTrials.gov: NCT03536182). The CIPHER-trial started in May 2019 and is supposed to be discontinued due to unknown issues.

To avoid gastrointestinal toxicity, it is crucial to define OAR constraints precisely. The α/β - value of the small intestine is not known exactly and there are differences in the literature. With an underlying assumed α/β - value of the upper gastrointestinal tract of 2–7 Gy, the constraints in the presented study protocol allow maximal 2 Gy equivalent doses (EQD2) of approximately 50–60 Gy in the upper gastrointestinal. Acceptable gastrointestinal toxicity could be observed in a SBRT study in pancreatic cancer accepting maximal doses up to 33 Gy in six fractions in the upper gastrointestinal tract (EQD 2 = 50–70 Gy, α/β = 2–7 Gy) by Herman et al. [23] Furthermore, Shinoto et al. observed acceptable toxicity rates allowing 46 Gy (RBE) (EQD2 = 55–65 Gy, α/β = 2–7 Gy) in the gastrointestinal tract when irradiating pancreatic cancer with carbon ions in 12 fractions [24]. Altogether, in our opinion, the presented dose constraints for the upper gastrointestinal are high but yet acceptable within a Phase II trial.

In the PACK-trial, we compare our in-house IMRT results of patients suffering from LAPC [14] with the data of Shinoto et al. [13]. Based on this comparison, we expect to considerably improve OS rates when irradiating with carbon ions. Here, we aim to evaluate this hypothesis in a small number of patients. Considering different pre-treatment schemes and the enrollment of both LAPC patients as well as locally recurrent pancreatic cancer patients, the study population will be relatively heterogenous. If the expected improvement will be validated, we intent to start a Phase III trial investigating carbon ion radiotherapy at HIT in comparison with modern IMRT. Comparison of carbon ion radiotherapy at different facilities is challenging [17]. Therefore, another aim of the present study is to establish carbon ion radiotherapy of localized non-metastasized pancreatic cancer at HIT.

### Trial status

Patient recruitment started in December, 2019.

## Data Availability

No unpublished datasets were used and/or analyzed for the study protocol. Any data is available from the corresponding author on reasonable request.

## Abbreviations

PACK: Pancreascarcinom und Kohlenstoffionenbestrahlung (pancreatic cancer and carbon ion radiotherapy)
LAPC: Locally advanced pancreatic cancer
RBE: Relative biological effectiveness
CA 19–9: Carbohydrate antigen 19–9
CEA: Carcinoembryonic antigen
HIT: Heidelberg Ion Beam Therapy Center
OAR: Organs at risk
DNA: Deoxyribonucleic acid
LET: Linear energy transfer
OS: Overall survival
LPFS: Local progression free survival
IMRT: Intensity-modulated radiotherapy
NCI: National Cancer Institute
CTC: Common terminology criteria
AE: Adverse events
EORTC: European Organisation for Research and Treatment of Cancer
QLQ: Quality of life questionnaire
ECOG: Eastern Co-operative Oncology Group
CT: Computed tomography
MRI: Magnetic resonance imaging
PET: Positron emission tomography
GTV: Gross tumor volume
CTV: Clinical target volume
ITV: Internal target volume
4D: Four-dimensional
PTV: Planning target volume
TPS: Treatment planning software
RT: Radiotherapy
LEM: Local effect model
EQD2: Equivalent dose at 2 Gy
BED: Biological equivalent dose
CR: Complete remission
PR: Partial remission
SD: Stable disease
PD: Progressive disease
RECIST: Response Evaluation Criteria In Solid Tumors
PFS: Progression free survival
DSMB: Data safety monitoring board
GCP: Good Clinical Practice
